# Long-read sequencing for cancer liquid biopsy: advancing precision oncology

**DOI:** 10.3389/fonc.2026.1811012

**Published:** 2026-06-10

**Authors:** Grace Guzman, Analiz Rodriguez

**Affiliations:** Department of Neurosurgery, College of Medicine, University of Arkansas for Medical Sciences, Little Rock, AR, United States

**Keywords:** cancer liquid biopsy, cfDNA (cell-free DNA), ctDNA (circulating tumor DNA), long-read sequencing (LRS), precision oncology

## Abstract

Liquid biopsy, which involves the study of tumor-derived genetic material shed into circulating body fluids, is a rapidly emerging minimally invasive approach for cancer diagnosis and monitoring. Most current cancer liquid biopsy workflows depend on short-read sequencing (SRS). However, SRS methods remain limited in their ability to detect and resolve structural variants (SVs), haplotype phasing, fusion transcripts, and epigenetic modifications. Long-read sequencing (LRS) technologies, including single-molecule real-time (SMRT) and nanopore sequencing, offer opportunities to overcome these limitations by preserving long-range molecular information and enabling multimodal characterization of tumor-derived material in biofluids. In this mini-review, we discuss the emerging role of LRS in cancer liquid biopsy, with primary emphasis on cell-free DNA (cfDNA) and circulating tumor DNA (ctDNA). We summarize recent studies using LRS-based liquid biopsy across multiple cancer types. Particular focus is placed on cancer types most actively investigated to date, such as lung, brain, and pediatric cancers, in which LRS-based liquid biopsy has shown promise in detecting SVs, methylation patterns, and tumor-of-origin (TOF) signals that may not be fully captured by SRS approaches. We also examine current technical and translational barriers of LRS in cancer liquid biopsy, such as pre-analytical variability, cost, and high computational demands. As sequencing technologies and analytical pipelines continue to advance, LRS is likely to serve as a complementary component of multimodal liquid biopsy strategies in precision oncology.

## Introduction

Liquid biopsy, the analysis of tumor-derived material from bodily fluids, has become an essential tool in precision oncology. This minimally invasive approach can include analytes such as cell-free DNA (cfDNA), circulating tumor DNA (ctDNA), circulating tumor cells (CTCs), extracellular vesicles, circulating RNA species, and other tumor-associated components. It allows characterization of cancer genomes and their evolution during treatment (1, 2). Compared to conventional tissue biopsy, liquid biopsy captures spatial and temporal tumor heterogeneity usually missed by single-site tissue sampling and permits serial collections for real-time molecular insights (3, 4).

Over the past decade, short-read next-generation sequencing (NGS) has been widely used for cancer liquid biopsy analysis. NGS provides sensitive detection of single-nucleotide variants (SNVs), small insertions/deletions (indels), point mutations, and methylation analysis in advanced cancers (5–8). The application of this short-read platform has proven clinical utility in identifying actionable mutations, tracking minimal residual disease, and guiding targeted therapy across multiple cancer types (9–14). Despite these successes, short-read (typically <600bp) sequencing remains restricted in characterizing complex genomic rearrangements, such as structural variants (SVs), and direct epigenetic modifications, which are increasingly recognized as promoters of oncogenesis, tumor heterogeneity, and therapeutic resistance (15, 16).

Long-read sequencing (LRS) offers opportunities to overcome many of these challenges (17). By producing contiguous reads exceeding several kilobases to megabases, LRS supports direct detection of SVs, long-range haplotypes, fusion transcripts, and full-length isoforms. These capabilities position LRS as a compelling strategy for advancing liquid biopsy toward comprehensive tumor genome and epigenome profiling. While application of LRS to liquid biopsy is still in early stages, emerging studies suggest its growing feasibility for cfDNA and ctDNA analysis. In this review, we summarize the current progress of LRS for cancer liquid biopsy, discuss its advantages and limitations, and outline future directions for its integration into precision oncology.

### LRS technologies relevant to cancer liquid biopsy

Recent efforts in cancer liquid biopsy have utilized two leading LRS platforms: single-molecule real-time (SMRT) sequencing from Pacific Biosciences (PacBio) and nanopore sequencing from Oxford Nanopore Technologies (ONT) (18, 19). Both platforms generate reads substantially longer than those produced by short-read NGS and support analyses such as SV detection, haplotype phasing and direct methylation profiling without bisulfite conversion (20–25). Specifically, direct methylation profiling without bisulfite conversion preserves fragment length and minimizes DNA loss, which is relevant to liquid biopsy because fragmented circulating nucleic acids may contain genomic and epigenomic features that are not fully captured by short-read methods. Although these features confer key advantages of LRS over short-read NGS, SMRT and nanopore sequencing differ fundamentally in read accuracy, throughput, and analytical trade-offs. A comparative summary of these two technologies is provided in **Table 1**.

**Table 1:** Comparison of Oxford Nanopore Technologies (ONT) and Pacific Biosciences (PacBio) long-read sequencing (LRS) platforms for cancer liquid biopsy applications.

Feature	ONT (Oxford nanopore technologies)	PacBio (Pacific biosciences)
Sequencing principle	Nanopore-based; DNA passes through a protein pore and current changes are measured in real-time	Single-molecule real-time (SMRT); fluorescently labeled nucleotides detected during polymerase synthesis in zero-mode waveguides
Typical read length	Ultralong reads (up to 2 Mb reported)	Long reads (typically ~10–25 kb for HiFi reads)
Accuracy profile	~92–97% (R9.4.1); improved accuracy with recent chemistries and basecalling (approaching >99% in optimized settings), but generally lower than HiFi reads	HiFi reads >99% base-level accuracy; highest consensus accuracy among LRS platforms
Error profile	Higher indel rates	Low error rates with reduced indels and mismatches
Throughput and scalability	Very high; PromethION can generate >100 Gb per flow cell; much higher total read counts per run	Lower throughput per run compared to ONT and less flexible in platform scalability
Turnaround time	Fastest; real-time data streaming; CNV and fragmentomic results achievable in 24 h from sample collection	Longer library prep and run times; not real-time; typically days for data generation
Native methylation detection	Direct detection of DNA methylation from native DNA without bisulfite conversion	Detection of base modifications via polymerase kinetics without bisulfite conversion
Strengths for liquid biopsy	Real-time sequencing, potential for integrated genomic and epigenomic profiling, and adaptability to fragmented analytes	High-accuracy variant detection, including SNVs and indels, with strong performance for consensus-based analyses
Key limitations for liquid biopsy	Error rates (though improving), need for optimized bioinformatics pipelines	Higher DNA input requirements, cost, and longer workflow times

Key technical features and analytical trade-offs between the two leading LRS platforms are summarized.

CNV, copy number variation; cfDNA, cell-free DNA; ctDNA, circulating tumor DNA; HiFi, high-fidelity; indel, insertion/deletion; NGS, next-generation sequencing; SNV, single-nucleotide variant; SV, structural variant.

In cancer research, SMRT and ONT nanopore sequencing have improved understanding of tumor architecture by uncovering variations previously undetected with short-read NGS. For example, using SMRT sequencing, investigators identified a complex *KLHDC2-SNTB1* fusion exceeding 10 kbp in the well-studied breast cancer cell line SK-BR-3 (26). Similarly, nanopore sequencing using the PromethION platform in lung adenocarcinoma and colorectal cancer revealed novel SVs and gene fusions (27, 28).

These studies demonstrate the capacity of LRS to identify rare but important oncogenic events that may be missed by traditional approaches. Beyond structural variants, LRS has captured characterization of copy number alterations and alternative splicing, including relevant copy number changes in CNS tumors and splice variants in pediatric medulloblastoma (29, 30). These studies highlight that LRS is reshaping cancer research by detecting novel, complex genomic and transcriptomic features central to tumor architecture and behavior. This progress establishes a strong foundation for its application in cancer liquid biopsy.

### Applications of long-read sequencing in cancer liquid biopsy

While liquid biopsy includes a variety of circulating analytes, current literature on LRS in cancer liquid biopsy focuses predominantly on cfDNA and ctDNA. Accordingly, this section emphasizes cfDNA and ctDNA applications of LRS while briefly discussing developing areas beyond these analytes.

Currently, short-read NGS and PCR-based assays constitute the primary modalities for detecting and analyzing cfDNA in liquid biopsy workflows (5, 26). Despite their significant impact, these assays are approaching their sensitivity and informational limits, particularly for tumor-of-origin inference and epigenomic profiling. These limitations contributed to the growing interest in LRS for liquid biopsy. Initial efforts to apply LRS platforms to ctDNA encountered technical limitations. For example, early evaluations of ONT-based sequencing for non-invasive prenatal diagnosis yielded unsatisfactory results due to low throughput and insufficient sensitivity for cfDNA fragments (31). These obstacles reflected a mismatch between standard LRS protocols, which are optimized for high-molecular-weight DNA, and the fragmented nature of cfDNA (5). To address this, researchers optimized standard protocols by adjusting bead clean-up volumes to retain short cfDNA fragments, employing updated sequencing chemistry and flow cells (SQK-LSK109 with R9.4.1), and combining low-coverage whole-genome sequencing with specialized segmentation algorithms (32). These modifications permitted successful detection of tumor-associated copy number variations (CNVs) in plasma samples with as few as ~2 million reads per sample (32). These advances established a foundation for tumor-specific long-read liquid biopsy applications.

**Figure 1:**
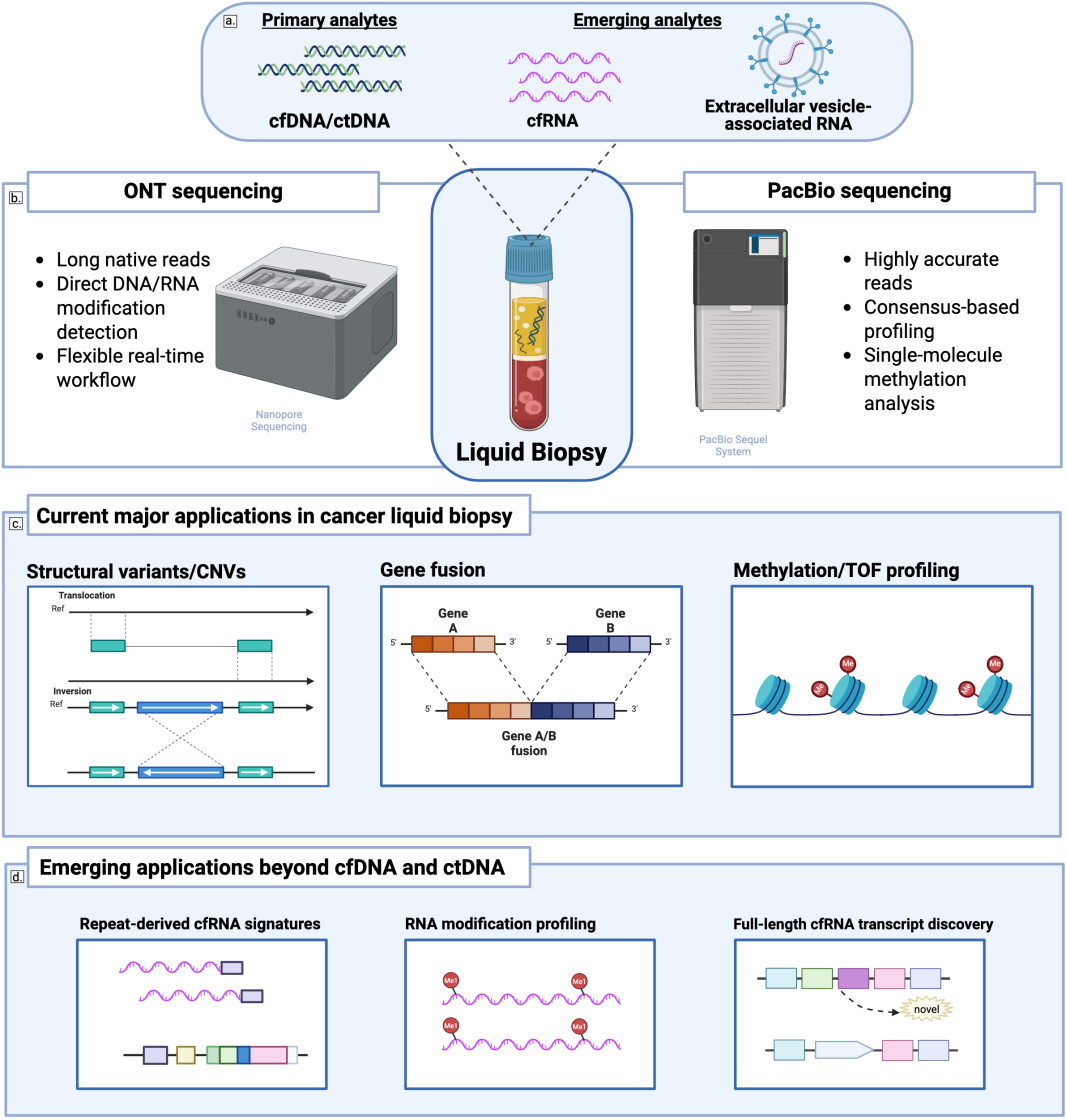
Expanding roles of long-read sequencing in cancer liquid biopsy. **(a)** Liquid biopsy analytes relevant to LRS in cancer. Current studies primarily emphasize cell-free (cfDNA) and circulating tumor DNA (ctDNA). Developing applications are exploring circulating cell-free RNA (cfRNA) and extracellular vesicle-associated RNA, which remain largely in proof-of-concept stages. **(b)** Overview of the two principal LRS platforms used in cancer liquid biopsy: Oxford Nanopore Technologies (ONT) and Pacific Biosciences (PacBio) single-molecule real-time (SMRT) sequencing. **(c)** Major applications of LRS in cancer liquid biopsy include structural variant (SV) and copy number variation (CNV) detection, gene fusion identification and methylation/tissue-of-origin (TOF) profiling among others. **(d)** Emerging applications alternative analytes include repeat-derived cell-free RNA (cfRNA) signature analysis, RNA modification profiling, and full-length cfRNA transcript discovery. Created in BioRender. Rodriguez, A. (2026) https://BioRender.com/bzajq7e.

Recent studies have challenged the assumption that cfDNA exists exclusively as short fragments. Using SMRT sequencing, two investigations identified long cfDNA in the plasma of pregnant women and cancer patients (33, 34). Notably, in a proof-of-concept study, Choy et al. applied single-molecule tissue-of-origin (TOF) analysis and found that a substantial fraction of plasma cfDNA existed as long molecules (>1 kb), with a median of 15.7% in hepatocellular carcinoma (HCC) patients, approximately 350-fold higher than that detected by an NGS platform (Illumina) (34). Although tumor-derived cfDNA was generally shorter and hypomethylated, fragments reached lengths of up to 13.6 kb, enabling single-molecule methylation profiling without bisulfite conversion. By examining methylation at multiple CpG sites on long cfDNA molecules, the authors developed an HCC-specific methylation score that significantly improved cancer discrimination (AUC, 0.91) relative to short cfDNA molecules (AUC, 0.75). These studies and ongoing improvements in LRS chemistry have increased interest in long-read based approaches for liquid biopsy in precision oncology (**Figure 1**).

Lung cancer represents one of the most actively investigated tumor types for LRS-based liquid biopsy (35, 36). Martignano et al. demonstrated that nanopore-based sequencing of cfDNA from lung cancer patients accurately detected tumor-associated CNVs previously linked to drug resistance (32). Subsequent investigations showed that ONT-based analysis achieved accuracy comparable to traditional short-read sequencing (35, 37). Katsman et al. also provided evidence that widespread DNA hypomethylation could serve as a general biomarker for ctDNA, offering a clear distinction between individuals with lung cancer and healthy controls. More recently, researchers developed a technique for detecting EGFR mutations using nanopore sequencing with blocker displacement amplification (BDA) (38). This targeted amplification method suppressed wild-type DNA and allowed detection of clinically actionable EGFR mutations even at low variant allele fractions. Furthermore, preliminary data from bronchoalveolar lavage (BAL) fluid showed the feasibility of nanopore long-read sequencing of native cfDNA in lung cancer patients utilizing as little as ~150 ng of input material (39). Remarkably, one-third of BAL cfDNA fragments exceeded 1 kb in length and were enriched for CpG methylation. This allowed simultaneous TOF profiling and detection of low-frequency tumor-associated somatic variants. Although these findings await peer-reviewed validation, they suggest tumor-proximal long-read liquid biopsy approaches could offer more comprehensive molecular tumor characterization and support longitudinal disease monitoring in lung cancer.

Central nervous system malignancies have also emerged as a leading tumor type for LRS-based cancer liquid biopsies. Cerebrospinal fluid (CSF) is the most relevant biofluid for liquid biopsies in central nervous system (CNS) malignancies due to its proximity to intracranial tumors. Multiple studies have shown that cfDNA concentrations in CSF are typically higher than in plasma and therefore provide superior sensitivity for liquid biopsy applications (40–43). In pediatric diffuse midline glioma, Bruzek et al. applied ONT sequencing (MinION) to CSF cfDNA and detected key histone mutations, H3F3A and H3C2, with 85% sensitivity and 100% specificity (44). Results were obtained within 12 hours and this workflow proved particularly valuable for non-invasive diagnosis and prediction of long-term clinical outcomes in populations where surgical sampling is limited or high risk. In medulloblastoma (MB), Filser et al. evaluated nanopore sequencing in the largest cohort to date, comprising 116 tumors (45). They found that nanopore-derived methylation and copy number profiles accurately classified >90% of cases into established molecular subgroups and subtypes when compared against gold-standard methylation assays (Illumina MethylationEPIC array). As of this review, this study represents the largest proof-of-concept that LRS can support more precise patient stratification for diagnosis.

Beyond lung and CNS malignancies, long-read cfDNA and ctDNA analysis has also shown promise in hematologic, hepatobiliary, colorectal, esophageal, and gynecologic malignancies. In pediatric B-cell acute lymphoblastic leukemia (B-ALL), long-read nanopore sequencing (MinION) was employed to analyze cfDNA from CSF, targeting clonal immunoglobulin heavy chain (IGH) VDJ rearrangements (46). IGH VDJ rearrangements are unique markers of clonality in B-cell malignancies and are important for monitoring disease progression in lymphoid malignancies (47, 48). Although the sample size was small (n=5), researchers successfully uncovered clonal heterogeneity, tracked minimal residual disease, and detected CNS involvement that escaped traditional diagnostic thresholds. This study highlighted the feasibility of LRS for liquid biopsy in hematologic malignancies and its potential to extend applications from mutation discovery to highly sensitive molecular monitoring for disease progression (49). While short-read NGS is the standard modality due to its well-validated performance, this study provides unique strengths of LRS for detecting complex or low-abundance events.

Additional studies in gastrointestinal and other solid tumors further illustrate the breadth of current cfDNA and ctDNA applications. Yu et al. used long plasma cfDNA to distinguish HCC from chronic hepatitis B virus (HBV) carriers through methylation-based TOF analysis (50). Building on this, another group constructed a reference methylome using nanopore-derived long cfDNA reads from CRC patients matched with immune cell profiles and then applied single-molecule classification to separate tumor-derived cfDNA from background DNA (51). Their method improved sensitivity for low tumor fractions, which addressed limitations of bulk cfDNA analyses where tumor signals are diluted. More recently, a proof-of-concept study introduced Nanopore Rolling Circle Amplification-enhanced consensus sequencing (NanoRCS) (52). NanoRCS uses rolling circle amplification of circularized cfDNA molecules with consensus calling to reduce sequencing errors and allow multimodal profiling. This technology integrated three signals, including SNVs, copy number alterations, and fragmentomic patterns, within a single assay. Importantly, the multimodal integration that NanoRCS provided reliably detected tumor fractions as low as 0.24% across esophageal adenocarcinomas, ovarian carcinoma, and granulosa cell tumors. These findings show the potential of LRS for multimodal liquid biopsy and offer a scalable strategy for individualized cancer monitoring.

### Emerging analytes beyond cfDNA and ctDNA

LRS-based cancer liquid biopsy studies remain largely focused on cfDNA and ctDNA. However, analytes beyond these, including circulating RNA and extracellular vesicle-associated RNA, may expand the scope of this technology. For example, Reggiardo et al. utilized single-molecule nanopore sequencing of plasma cell-free RNA (cfRNA) to identify cancer-associated repeat-derived RNA signatures across multiple solid tumors, including pancreatic, liver, and esophageal cancers (53). In another study, single-molecule nanopore profiling of circulating RNA detected lung cancer-associated 2’-O-methylation pattern in blood. This study highlighted that RNA epitranscriptomic profiling could serve as an additional biomarker layer beyond sequencing information alone (54). In a proof-of-concept preprint, Peddu et al. showed that long-read nanopore sequencing of full-length cfRNA in patients with Barrett’s esophagus with high-grade dysplasia and esophageal adenocarcinoma revealed a much broader transcriptome than captured with traditional approaches. Researchers found more than 250,000 novel intergenic cfRNAs and built a custom transcriptome reference and machine-learning framework that classified precancerous and cancer states (55). Overall, these studies suggest that other analytes may broaden future LRS-based liquid biopsy applications, although current evidence remains early-stage and substantially less defined than existing than cfDNA and ctDNA workflows.

## Discussion

### Clinical promise of LRS in cancer liquid biopsy

LRS overcomes key barriers of short-read sequencing and may offer a more comprehensive tumor profiling (19). Its features of improved detection of SVs, direct methylation profiling, and multimodal characterization of DNA positions long-read platforms as a potential great utility for precision oncology. However, current evidence remains strongest for LRS for cfDNA and ctDNA-based applications and it is still unclear which tumor types will ultimately derive the greatest benefit from long-read approaches over short-read assays.

### Current technical and biological barriers

Despite these advantages, several challenges hinder widespread clinical adoption of LRS for cancer liquid biopsy. This limitation is particularly true in early-stage disease or tumors with minimal vascular shedding, where ctDNA may constitute less than 0.1% of total ctDNA (43, 56). Consequently, low tumor fractions can reduce the sensitivity of current long-read platforms relative to short-read NGS. The reduction of analytical sensitivity may not be fully justified even with broader molecular information. Additionally, cfDNA originates from a heterogeneous mixture of tumor and non-tumor cell populations, and intratumor heterogeneity may complicate quantification of fragments containing informative somatic alterations (42, 57, 58). Because not all cfDNA molecules, including long fragments, encode tumor-specific signals, variant allele fractions must be interpreted with caution. Sensitive analytical frameworks are needed to distinguish low-level tumor-derived signal from abundant background DNA.

### Pre-analytical and workflow limitations

Pre-analytical variability further impedes long cfDNA analysis. Variables including blood collection tubes, processing time, storage conditions, and cfDNA extraction protocols can impact the integrity of long cfDNA fragments, potentially introducing systematic bias into downstream fragmentomic, methylation, and structural analyses (59–61). Unlike short-read NGS methods, which have benefited from years of protocol optimization, LRS-based liquid biopsy approaches lack broadly accepted pre-analytical guidelines. This is important because the performance of long-read assays may partly reflect differences in sample handling rather than intrinsic sequencing capability alone. The development of reproducible workflows for long cfDNA preservation and processing will be essential to minimize inter-study variability, facilitate cross-institutional collaboration and integrate into oncological care (62). Additional barriers include ethical and regulatory considerations related to genomic data handling, informed consent, and patient privacy, though discussion of these issues is beyond the scope of this review (63).

### Platform-specific and computational considerations

While recent advancements have significantly increased sequencing accuracy of both PacBio and ONT workflows, analytical trade-offs persist between platforms (18, 64–66). A comparative study of real-time detection of long cfDNA in plasma found that PacBio HiFi sequencing produced significantly fewer single-nucleotide and small indel errors than ONT-based assemblies. The latter generated an average of approximately 1.06 errors per kb and was associated with downstream annotated artifacts, including truncated protein predictions (65). In contrast, ONT produced ultralong reads (up to 2 Mb), thus maximizing assembly contiguity and resolving repetitive regions. Subsequent research showed that PacBio datasets tend to contain a higher proportion of long cfDNA fragments and superior per-base accuracy, whereas ONT sequencing may yield two- to four-fold more fragments eligible for downstream TOF, although it requires additional preprocessing steps (50). These differences may influence platform selection based on study design and clinical applicability. For example, studies that prioritize confident base-level interpretation may benefit from higher-accuracy workflows, whereas those centered on fragment length or broader structural features may better suited for ONT technologies. As a result, broader clinical implementation will likely require greater standardization for platform selection than is currently available.

### Cost and translational feasibility

Finally, cost and data storage further influence the translational feasibility of LRS. While sequencing expenses are declining, LRS remains substantially more expensive than short-read platforms. Costs are even greater when high-depth or multimodal profiling is required (67). Additionally, long-read assays produce large data volumes that necessitate significant computational infrastructure for processing and long-term storage, further increasing overall costs (68). To address these challenges, data compression techniques and alternative storage formats are being engineered to reduce storage requirements and improve the efficiency of LRS analysis (69). These cost considerations are important because LRS-based liquid biopsy adoption may not only depend on technical superiority but also whether this method provides enough added value to justify its high costs and complexity.

### Future directions

The future of LRS in cancer liquid biopsy will depend on continued advances in sequencing chemistry, computational analysis, and assay design. Emerging studies suggest that machine learning and artificial intelligence (AI) tools may improve basecalling accuracy, multimodal integration, and longitudinal interpretation of cfDNA (70–72). However, while these advances are promising, the most likely role for LRS may be as a complement, rather than a replacement, to short-read assays. The strengths of LRS platforms may be integrated through hybrid sequencing strategies. These approaches leverage pairing long-range genomic content from LRS with high per-base accuracy of short-read NGS, resulting in more precise tumor profiling (73, 74). Such hybrid workflows have also been proposed as strategies to mitigate high costs and performance limitations associated with LRS alone (75–77).

## Conclusion

Long-read sequencing is beginning to reshape the landscape of cancer liquid biopsy. These technologies capture SVs, long-range haplotypes, and epigenetic features from tumor-derived fragments and provide biological context that short-read platforms cannot achieve alone. Currently, however, the bulk of LRS-based cancer liquid biopsy remains focused on cfDNA and ctDNA analytes, while studies involving other analytes are still comparatively early in development. While important barriers persist regarding sensitivity, standardization, and cost, ongoing computational and analytical innovations are addressing these limitations. As these technologies become more refined and cost-effective, LRS is likely to be an important complementary component of multimodal liquid biopsy strategies for personalized cancer care.
